# Effectiveness of Electroconvulsive Therapy in the Remission of Malignant Catatonia Associated With Schizophrenia: A Case Report

**DOI:** 10.1155/crps/8478463

**Published:** 2026-01-29

**Authors:** Lucas Reis Alves Mota, Joyce dos Santos Neves, Carolina de Souza Baldin, Gustavo Barros da Silva, Gustavo Bigaton Lovadini, Silvia Cristina Mangini Bocchi

**Affiliations:** ^1^ Botucatu School of Medicine, São Paulo State University “Júlio de Mesquita Filho” (UNESP), Botucatu, State of São Paulo, Brazil, unesp.br; ^2^ Botucatu Clinical Hospital, Botucatu, State of São Paulo, Brazil; ^3^ Integrated Health Care Center “Professor Cantídio de Moura Campos”, Botucatu, State of São Paulo, Brazil

**Keywords:** case reports, catatonia, electroconvulsive therapy, psychiatry, schizophrenia

## Abstract

Despite the robust body of evidence supporting the efficacy and safety of electroconvulsive therapy (ECT) in the treatment of severe psychiatric disorders, this procedure remains at the center of social debate, often influenced by historical prejudice. Such misconceptions have contributed to restricted access, which typically occurs either through costly private practice or in large academic centers within the public health system. Among the clinical conditions for which ECT is considered a first‐line treatment, malignant catatonia stands out—a rare and potentially fatal syndrome characterized by psychomotor disturbances and severe autonomic instability. If not treated promptly, its mortality rate may reach up to 50%. This case report describes the successful treatment of malignant catatonia in a patient with schizophrenia, in whom ECT sessions were administered intermittently and without a subsequent maintenance phase during inpatient care at a specialized psychiatric hospital affiliated with the Unified Health System in the state of São Paulo, Brazil. After 10 sessions, there was complete remission of the catatonic state, followed by hospital discharge for outpatient follow‐up. After 16 months, the patient maintained psychiatric stability, medication adherence, and partial independence in daily activities, with no new episodes of psychiatric decompensation. The case underscores the effectiveness of ECT in achieving remission of a rare and potentially life‐threatening disorder.

## 1. Introduction

Electroconvulsive therapy (ECT) is a safe and effective treatment for a range of psychiatric disorders [1]. The procedure is performed under general anesthesia, during which seizures are artificially induced by applying electrical currents—with predefined amplitude, intensity, and duration—to specific brain regions [2].

Although its exact mechanism of action has not yet been fully elucidated, substantial evidence exists regarding its influence on neurobiology [3, 4]. The best‐documented effects include modulation of monoaminergic neurotransmitters [5–8], alterations in the hypothalamic‐pituitary‐adrenal axis [8], anticonvulsant properties [9], and the induction of neurogenesis with consequent changes in neuronal plasticity [8].

Numerous studies have informed protocols for the treatment of mental and, in some cases, neurological disorders. When planning ECT, several factors must be considered, including the characteristics and severity of the underlying psychiatric condition, the patient’s overall medical status, and their response to treatment.

Scientific evidence supports administering three sessions per week on alternate days for 2 – 4 weeks for most clinical indications [1, 10]. For more severe or treatment‐resistant cases, daily sessions over two to three consecutive days are recommended, a method known as “en bloc” ECT [11–16]. Twice‐weekly administration has also been shown to be effective, although it is associated with a longer total treatment duration, which supports the preference for the three‐sessions‐per‐week regimen adopted by most centers [17].

Beyond debates about its efficacy, ECT continues to be the subject of intense social discussion, including within the scientific community [10, 18]. In Brazil, access to ECT remains largely restricted to major public health centers or private services, where costs are high [19].

Among the various disorders that may benefit from ECT, malignant catatonia is particularly noteworthy—a condition for which ECT is considered the treatment of choice [13–15, 20].

Catatonia is a clinical syndrome characterized by a wide range of psychomotor abnormalities, including spastic rigidity, immobility, mutism, negativism, waxy flexibility, stereotypies, and automatic obedience [21]. First described in 1874 by German psychiatrist Karl Ludwig Kahlbaum, catatonia remains a subject of intense debate within psychiatry [22]. Although no consensus exists regarding its prevalence, several epidemiological studies estimate it to be ~10% in acute psychiatric cases [21, 22].

Until the publication of ICD‐11, catatonia was not considered an independent nosological entity and was recognized as secondary to various clinical and psychiatric conditions—most commonly bipolar disorder and schizophrenia [13].

In 1934, the German psychiatrist Karl Heinz Stauder first described cases of catatonia associated with febrile states and extreme agitation, which could progress to severe stupor, coma, and death. This variant became known as Stauder’s lethal catatonia, pernicious catatonia, or malignant catatonia [22].

Malignant catatonia is a rare syndrome of undetermined prevalence, characterized by typical catatonic symptoms combined with severe autonomic instability, with mortality rates ranging from 20% to 50% if not treated within the first days of onset [13, 20, 23]. Accordingly, the literature recommends the use of “en bloc” ECT when available [13–15].

This case report describes the successful treatment of malignant catatonia in a patient with schizophrenia using intermittently administered ECT, without a subsequent maintenance phase, during inpatient care at a specialized psychiatric hospital in São Paulo, Brazil.

The significance of this case is underscored by the low prevalence and high morbidity and mortality of malignant catatonia, whose primary treatment—although highly effective and safe–still faces substantial barriers to implementation and remains largely inaccessible to most users of the Brazilian healthcare system.

## 2. Case Presentation

### 2.1. Patient Information

A 56‐year‐old white male, single and without children, with 8 years of formal education, living long‐term with two brothers, one of whom serves as his legal guardian. He has a history of systemic arterial hypertension and type II diabetes mellitus under treatment, as well as a family psychiatric history, including a brother diagnosed with schizophrenia.

The patient has had paranoid schizophrenia (ICD‐10: F20.0) since 1989, with six psychiatric hospitalizations to date. Following his fourth hospitalization in 2002, he remained psychiatrically stable for 20 years on thioridazine. In September 2022, after the medication became unavailable, he developed behavioral disorganization and aggression, requiring a 29‐day inpatient admission. He was discharged on October 10, 2022.

He maintained good symptom control until December 28, 2023, when he exhibited a sudden behavioral change (mutism), with no organic cause identified after laboratory investigations and cranial computed tomography, which revealed only mild global atrophy and slight atheromatosis. At that time, he was receiving a daily regimen of quetiapine 100 mg, levomepromazine 100 mg, and clonazepam 2 mg, with reports of irregular adherence.

**Figure 1:**
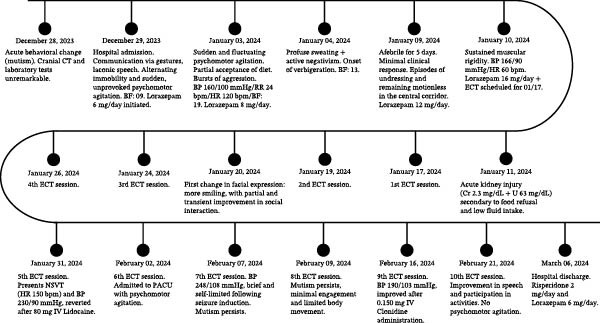
Clinical course during hospitalization, including psychiatric symptoms, Bush–Francis Catatonia Rating Scale scores, pharmacological management, electroconvulsive therapy sessions, and major clinical events. CT, computed tomography; BF, Bush–Francis Catatonia Scale; mg/d, milligrams per day; BP, blood pressure (measured in millimeters of mercury, mmHg); RR, respiratory rate (measured in breaths per minute); HR, heart rate (measured in beats per minute); ECT, electroconvulsive therapy; Cr, serum creatinine (mg/dL); U, urea (mg/dL); NSVT, non‐sustained ventricular tachycardia; IV, intravenous; PACU, post‐anesthesia care unit.

The patient’s clinical course during hospitalization is summarized in Figure 1.

### 2.2. Clinical Findings and Diagnostic Assessment

The patient was admitted to a psychiatric hospital on December 29, 2023, presenting with behavioral disorganization, mental confusion, global disorientation, and mutism. Initial psychiatric evaluation revealed disorganized motor behavior, alternating between periods of immobility and psychomotor agitation. An initial hypothesis of catatonia secondary to schizophrenia was considered supported by the Bush–Francis Catatonia Rating Scale (BFCRS score = 9), and lorazepam 6 mg/day was initiated. Laboratory tests showed a severe elevation in creatine phosphokinase (CPK 3.350 U/L – Reference Range: 24–195 Units/L), attributed to muscular rigidity (Figure 1).

Over the following 2 weeks, he developed predominant mutism, alternating with laconic speech or verbigeration. He exhibited muscular rigidity and stereotyped movements, along with grimacing, waxy flexibility, and negativism. His gaze was perplexed, and he remained hypervigilant, with sudden episodes of aggression and unprovoked escape attempts. Physical examination revealed intermittent sweating and autonomic instability, characterized by hypertensive peaks (BP 140/80 to 170/140 mmHg), febrile/subfebrile episodes (37.5 to 38.3°C), and tachycardia (HR 100–130 bpm).

Due to progressive clinical deterioration, the daily lorazepam dose was gradually increased: 8 mg on January 3, 2024; 12 mg on January 9, 2024; and 16 mg on January 10, 2024 (Figure 1). Considering this course, the diagnostic considerations were revised to malignant catatonia (BFCRS score = 19) and/or neuroleptic malignant syndrome (NMS), according to consensus diagnostic criteria established by an expert panel [24].

Following initial benzodiazepine optimization, vital signs improved, with resolution of febrile and hypertensive peaks. However, psychiatric and motor abnormalities persisted, including mutism, immobility, fixed gaze, catalepsy, and psychomotor agitation, with only modest improvement on the BFCRS score = 13 (Figure 1).

Despite encouragement of fluid intake, after approximately 2 weeks of hospitalization, the patient developed prerenal acute kidney injury (creatinine 2.3 mg/dL, urea 66 mg/dL), which was successfully treated with intravenous hydration. During follow‐up, no clinical or laboratory findings suggested an infectious process, nor were any abnormalities observed in other requested tests (complete blood count, thyroid function, electrolytes, or liver function).

Although Intensive Care Unit (ICU) admission was clinically indicated, this resource was unavailable. Due to the lack of an available bed, immediate initiation of ECT was also not possible. Consequently, antipsychotics—previously used for chemical restraint during psychomotor agitation—were discontinued, and supportive care was implemented, including vital sign monitoring, head‐of‐bed elevation, measures to maintain fluid balance, and assisted feeding. Hypertensive peaks were managed with oral antihypertensive therapy, and dipyrone was administered to control hyperthermia.

### 2.3. Therapeutic Intervention

Approximately 17 days after admission, on January 17, 2024, ECT was initiated following slight and partial improvement of the catatonic state with pharmacological treatment. Two weekly sessions (Wednesdays and Fridays) of bitemporal ECT were administered over 5 weeks, totaling 10 sessions (Figure 1). This schedule was determined by limitations in operating room availability at the treating facility and the availability of the two qualified professionals required to perform the procedure.

Lorazepam was discontinued 24 h prior to each session to minimize its anticonvulsant effect and was reintroduced immediately afterward. Before initiating ECT, risperidone 2 mg/day was prescribed to assist with behavioral management.

Throughout the ECT treatment period, multiple serial reassessments were conducted. Gradual improvement in catatonic symptoms was observed, with more sustained progress noted after the seventh session. The decision to forgo maintenance sessions was based on the clinical severity of other patients on the waiting list and the fact that the facility had only one ECT device.

All sessions were performed using a MECTA 5000Q device. The average duration of the procedure, excluding anesthetic induction and time in the post‐anesthesia care unit (PACU), was 3.2 min. Neuromuscular blockade was achieved with succinylcholine in all sessions, supported by literature evidence due to its rapid onset and short half‐life (typically under 10 min). Succinylcholine was chosen over rocuronium because of limited access to the latter at the facility and because febrile symptoms had resolved by the time ECT was initiated. Etomidate was used as the anesthetic inducer, as supported by literature for its association with prolonged seizure duration and a favorable cardiovascular safety profile [2]. Generalized seizure duration ranged from 23 to 72 s in all sessions.

Of the 10 sessions performed, six were uneventful. During the fifth session, the patient developed stable, non‐sustained ventricular tachycardia (HR: 150 bpm) associated with a hypertensive peak (230/90 mmHg), which reverted to sinus rhythm after 80 mg IV lidocaine. In the sixth session, the patient was admitted to the PACU with psychomotor agitation, requiring physical restraint for ~20 min. During the seventh session, a brief, self‐limited hypertensive peak (248/108 mmHg) occurred immediately after seizure induction. In the ninth session, another hypertensive peak (190/103 mmHg) was managed with 0.150 mg IV clonidine, achieving blood pressure control (136/77 mmHg).

The baseline ECT session protocol is summarized in Table 1.

**Table 1:** The baseline ECT session protocol.

N°	Date	Start	End	Device	Charge (mC)	Amplitude (m/s)	Frequency (Hz)	Duration (s)	Current (A)	Energy (J)	Impedance (omhs ‐ Ω)	Seizure duration (s)	Electrode placement	Anesthetic (etomidate)	Muscle relaxant (succinylcholine)
1	17/01	07h58	08h00	MECTA 5000Q	120	0,5	60	2,5	800	21,1	400/173	65	Bitemporal	20 mg	100 mg
2	19/01	07h29	07h35								560/203	58			
3	24/01	07h40	07h45								520/196	63			
4	26/01	07h40	07h45								480/214	72			
5	31/01	07h43	07h46								460/195	23			
6	02/02	07h38	07h40								390/180	40			
7	07/02	07h45	07h47								510/224	23		25 mg	
8	09/02	07h30	07h33								730/204	50		20 mg	
9	16/02	07h50	07h51								640/183	48		25 mg	
10	21/02	07h32	07h35								1840/218	25		25 mg	

### 2.4. Follow‐up and Intervention Outcomes

After 10 ECT sessions, complete remission of mutism and previously observed motor abnormalities was achieved. The patient remained calm, cooperative, and communicative, with improved auto‐ and allopsychic orientation. There was full resolution of motor rigidity, perplexed gaze, sudden and fluctuating episodes of psychomotor agitation, and escape attempts. For the first time since admission, the patient’s thought content became accessible, expressing a desire for discharge and to be with family. He ultimately scored zero on the BFCRS.

In this clinical context, he was discharged on March 6, 2024, for continuation of outpatient treatment, on a daily regimen of risperidone 2 mg, lorazepam 6 mg, and atenolol 100 mg. At the time of discharge, his systemic blood pressure remained between 120–130 × 80–90 mmHg (Figure 1).

Sixteen months after discharge, a synchronous videoconference was conducted with the patient’s caregiver for an interview. At that time, it was reported that the patient had experienced no further episodes of acute exacerbation of his underlying psychiatric disorder, maintaining psychiatric stability and adequate adherence to continuous medication (risperidone 2 mg/day) and outpatient care. The caregiver also reported that the patient retained partial independence in basic activities and certain instrumental activities, such as visiting nearby grocery stores and bakeries. The family member emphasized that the treatment provided was of fundamental importance for the patient’s improvement.

## 3. Discussion

ECT remains a highly controversial treatment in psychiatry and mental health [25]. Although regarded as one of the safest and most effective interventions for a range of severe mental disorders [26], its portrayal in the media and public perception continues to echo an era when psychiatric practice itself was misused as a tool for torture, punishment, and social control [10, 27].

Advances in medical science—particularly in anesthesiology and medical physics—have markedly distanced modern ECT from the way it was practiced between the 1930s and 1950s, when it was associated, among other problems, with bone fractures and significant anticipatory fear [26]. These developments have enabled ECT to become one of the safest procedures performed under general anesthesia [2].

Another major challenge ECT continues to face is its globally limited availability within public and private healthcare systems, where it is considered a highly specialized treatment of exceptionally rare prevalence [28]. In this respect, Brazil is no exception. Data indicate that ECT is available only in major public centers of the Unified Health System, predominantly university hospitals [19], or through private care, either out‐of‐pocket or via insurance coverage.

Among the psychiatric syndromes and disorders for which ECT has demonstrated efficacy are treatment‐resistant major depressive disorder, active suicidal ideation, acute mania, and schizophrenia [10]. Of these, one condition warrants particular attention: malignant catatonia.

Catatonia is a semi‐volitional motor syndrome first formally described in medical literature in 1874 by psychiatrist Karl Ludwig Kahlbaum—although a remarkably similar description can be found more than 20 years earlier in Herman Melville’s literary work *Bartleby*, *the Scrivener*. While it is not recognized as a distinct nosological entity in DSM‐5‐TR (2022), the ICD‐11 does classify it as such [29], with diagnostic criteria outlined in Table 2 [29].

**Table 2:** Features of catatonia in ICD‐11 and DSM‐5‐TR [29].

Clinical feature	Descriptions^a^	ICD‐11	DSM‐5‐TR
Decreased psychomotor activity			
Starting	Fixed gaze, decreased blinking	X	—
Ambitendency	Appears “motorically stuck” in indecision	X	—
Negativism	Opposing or behaving contrary to requests	X	X
Stupor	No or markedly reduced psychomotor activity	X	X
Mutism	No or very little verbal response (excludes other conditions affecting speech)	X	X
Increased psychomotor activity			
Extreme hyperactivity	May include agitation or extreme emotions	X	X
Impulsivity	Sudden, unprovoked, inappropriate behavior	—	—
Combativeness	Undirected striking out against others	—	—
Abnormal psychomotor activity			
Grimacing	Odd or distorted facial expressions	X	X
Mannerisms	Odd but purposeful movements	X	X
Posturing	Spontaneous maintenance of a posture held against gravity	X	X
Stereotypy	Repetitive, non‐goal‐directed motor activity	X	X
Rigidity	Resistance with increased muscle tone	X	—
Echolalia	Mimicking examiner’s speech	X	X
Echopraxia	Mimicking examiner’s movements	—	X
Verbigeration	Repetition of words, phrases, or sentences	X	—
Waxy flexibility	Slight, even resistance to positioning	X	X
Catalepsy	Passive induction of a posture held against gravity	X	X

Abbreviations: DSM, diagnostic and statistical manual of mental disorders; ICD, international statistic classification of diseases and related health problems.

^a^Abridged.

Malignant catatonia represents a clinical variant in which catatonia is accompanied by signs of autonomic instability, including hyperthermia as well as changes in blood pressure, heart rate, and respiratory rate [30]. Mortality rates range from 20% to 50% in inadequately treated populations [30, 31]. Current first‐line treatments include lorazepam and ECT [30].

The use of antipsychotics in catatonia remains one of the most controversial aspects of its management [16, 32]. Although these medications have been employed for decades, particularly in catatonia associated with primary psychotic disorders, the evidence is inconsistent, and there is substantial concern regarding the potential for clinical deterioration, including the induction of malignant catatonia or NMS [16, 32, 33].

Several authors recommend postponing antipsychotic initiation until the catatonic episode has resolved or introducing them only after stabilization with benzodiazepines [32]. This position is based on reports of ineffectiveness or clinical worsening when antipsychotics are started early, as well as series describing progression from catatonia to NMS following the use of first‐generation antipsychotics [32].

The relationship between antipsychotics, catatonia, and malignant catatonia is particularly more complex. The literature consistently highlights the difficulty in distinguishing NMS from malignant catatonia, especially in the context of antipsychotic exposure [16]. Some authors even conceptualize NMS as a toxic or iatrogenic subtype of malignant catatonia induced by antipsychotics [16]. Consequently, antipsychotics should be avoided in patients exhibiting signs of malignant catatonia or NMS.

Despite these concerns, there are also reports of therapeutic benefit. In recent years, an increasing number of successful cases have been described involving second‐generation antipsychotics (SGAs) in non‐malignant catatonia [32]. In catatonia associated with schizophrenia, for example, it has been hypothesized that antipsychotics may ameliorate the catatonic state by treating the underlying psychotic disorder [16, 32]. Still, the evidence base is scarce and low quality [16].

For these reasons, in cases when antipsychotics are deemed necessary, typically catatonia in the context of a primary psychotic illness, experts recommend the use of low‐potency second‐generation antipsychotics (SGAs), ideally co‐administered with a benzodiazepine, to reduce the risk of clinical worsening [16, 33]. These agents appear to carry a lower probability of precipitating worsening of symptoms, although the supporting evidence remains limited.

In this patient’s case, it was observed that the catatonia emerged following the abrupt discontinuation of his continuous psychotropic medications. The management of psychomotor agitation was carried out primarily with intramuscular midazolam. Risperidone (an SGA) was introduced a few days prior to the initiation of ECT, based on the clinical judgment that controlling the underlying psychiatric condition could positively influence the course of the catatonia while the patient was concurrently receiving high doses of lorazepam.

The presented case is particularly significant as it underscores the near absence of access to this treatment within the healthcare system: despite meeting priority clinical criteria, technical and logistical limitations delayed treatment initiation until 7 days after the clinical decision, with sessions administered at intervals of two to 5 days.

Moreover, given that the time between diagnosis and ECT initiation is directly associated with survival rates [21], it is noteworthy that the patient achieved full remission of catatonia after only 10 sessions, with no recurrence of symptoms even in the absence of maintenance therapy.

This case report therefore reinforces the existing literature by demonstrating the efficacy and safety of ECT [1, 2, 11, 14–16, 21, 28, 30], while at the same time emphasizing the need to expand access to this intervention within Brazil’s Unified Health System and to invest in the training of professionals qualified to perform the procedure.

## Author Contributions

Lucas Reis Alves Mota and Joyce dos Santos Neves conceived the case report. Carolina de Souza Baldin collected and selected the data. Gustavo Barros da Silva prepared the initial draft. Lucas Reis Alves Mota wrote and developed the manuscript. Joyce dos Santos Neves and Gustavo Bigaton Lovadini critically revised the text. Silvia Cristina Mangini Bocchi supervised the entire process and contributed at all stages.

## Funding

This manuscript received CAPES resources (AUXPE number 3703/25).

## Ethics Statement

The case report project was approved by the Research Ethics Committee of the Botucatu Medical School – UNESP on August 19, 2025 (CAAE: 86504225.7.0000.5411, Official Letter Number 7.774.699). Video‐recorded informed consent was obtained from the patient’s brother, acting as legal guardian and family caregiver.

## Conflicts of Interest

The authors declare no conflicts of interest.

## Data Availability

The data supporting this case report are not available in order to protect patient anonymity.
